# Patient Perceptions of Open, Laparoscopic, and Robotic Gynecological Surgeries

**DOI:** 10.1155/2016/4284093

**Published:** 2016-10-20

**Authors:** Mohamad Irani, Cheruba Prabakar, Sepide Nematian, Nitasha Julka, Devika Bhatt, Pedram Bral

**Affiliations:** ^1^The Ronald O. Perelman and Claudia Cohen Center for Reproductive Medicine, Weill Cornell Medicine, New York, NY 10021, USA; ^2^Department of Obstetrics and Gynecology, Maimonides Medical Center, Brooklyn, NY 11219, USA

## Abstract

*Objective*. To investigate patient knowledge and attitudes toward surgical approaches in gynecology.* Design*. An anonymous Institutional Review Board (IRB) approved questionnaire survey.* Patients/Setting*. A total of 219 women seeking obstetrical and gynecological care in two offices affiliated with an academic medical center.* Results*. Thirty-four percent of the participants did not understand the difference between open and laparoscopic surgeries. 56% of the participants knew that laparoscopy is a better surgical approach for patients than open abdominal surgeries, while 37% thought that laparoscopy requires the surgeon to have a higher technical skill. 46% of the participants do not understand the difference between laparoscopic and robotic procedures. 67.5% of the participants did not know that the surgeon moves the robot's arms to perform the surgery. Higher educational level and/or history of previous abdominal surgeries were associated with the highest rates of answering all the questions correctly (*p* < 0.05), after controlling for age and race.* Conclusions.* A substantial percentage of patients do not understand the difference between various surgical approaches. Health care providers should not assume that their patients have an adequate understanding of their surgical options and accordingly should educate them about those options so they can make truly informed decisions.

## 1. Introduction

Minimally invasive surgery is the standard of care in the surgical management of many gynecologic conditions and is a modality commonly offered during treatment planning [1–5]. There are several readily available sources of information, such as brochures, pamphlets, and online resources, which can provide patients with basic information about minimally invasive options. However, despite these resources and the frequent use of minimally invasive techniques, there are few data that demonstrate patients' understanding of the advantages, disadvantages, and appropriate indications for their use [6]. Furthermore, the process of providing information in a comprehensible manner presents a major barrier for most clinicians. While several factors may be expected to influence a patient's degree of understanding (e.g., education and previous experience with a particular surgical approach), no study to date has looked at patients' perceptions and the factors that influence their understanding of various surgical approaches.

The purpose of the study was to determine if patients who seek obstetrical or gynecological care recognize basic differences between open, laparoscopic, and robotic-assisted laparoscopic surgery. The study also aimed to explore factors that affect patients' knowledge about surgical approaches in gynecology.

## 2. Methods and Materials 

Women who presented between January and September 2014 for routine obstetrical and gynecological care in two separate sites in Brooklyn, New York, were offered participation in the study. Participants completed a questionnaire in the obstetrics and gynecology (OBGYN) clinic at Maimonides Medical Center and at a private office affiliated with the hospital. Maimonides Medical Center is a nonprofit, nonsectarian 711-bed community hospital. The community served by the hospital and the private office is ethnically and culturally diverse.

The questionnaire consists of eight items (see the Appendix). The survey items included demographic information and questions that asked patients to rate their degree of familiarity with different surgical modalities (open, laparoscopic, and robotic-assisted laparoscopic surgery) or asked them specific questions about the difference between one method and another. The answers were based on a Likert scale. The survey was first piloted among ten patients to obtain feedback and ensure understanding and clarity of the questions prior to finalizing the instrument. Surveys with 80% or greater completion were included for analysis.

Results were described as frequency (percent) when responses were coded as categorical variables (e.g., ethnicity, previous surgery, and education) and outcomes were based on a Likert scale. Tests to determine predictors of either knowledge of, or familiarity with, the surgical approaches were carried out using Chi-square or Fisher's exact test. Odds ratio (OR) with 95% confidence intervals (CI) was calculated and adjusted for all identified confounders. A sample size of 194 was deemed necessary to detect a correlation of 0.2 between the participant's educational level and her knowledge about various surgical techniques, with 5% level of significance and 80% power. Assuming that 20% of participants might not answer ≥80% of the questions, the aim was to recruit 242 patients. The significance level was <0.05 for all comparisons. All analyses were performed using STATA statistical software version 14 (StataCorp LP).

## 3. Results

The survey was offered to 273 patients; 242 (88.6%) agreed to participate. Out of the 242 recruited patients, a total of 219 completed the survey (answered ≥80% of the questions): 145 from the clinic and 74 from the private office. Fifty-six percent of the participants presented for obstetrical care while 44% were seeking gynecological care. The demographic characteristics are summarized in Table 1 showing the diversity in the age, race, and educational level of the included patients. Approximately 10% of the participants underwent previous abdominal surgery other than cesarean section. Forty-six percent of the surveyed women do not have college degree.

One-third of the participants (34%) did not understand the difference between open and laparoscopic surgeries. Age and race were not significant predictors of patient's knowledge to differentiate between open and laparoscopic procedures (*p* = 0.07; *p* = 0.46, resp.). However, the most educated participants (college graduate/professional) had a significantly better understanding of the difference between open and laparoscopic surgeries than the least educated participants (less than high school) (80% versus 43.4%, *p* = 0.007; aOR = 4.5, 95% CI: 1.5–13.9) after adjusting for age and race. Similarly, participants who underwent abdominal surgeries understood this difference better than those who did not (100% versus 62%, *p* < 0.001).

Fifty-six percent of the participants knew that laparoscopy is a better surgical approach for patients than open abdominal surgeries, while 37% thought that laparoscopy requires the surgeon to have a higher technical skill. Highest level of education was associated with a higher rate of correct answers of these two questions than lowest level of education (78% versus 13%, *p* < 0.001; aOR = 12.7, 95% CI: 3.5–46.4 and 60% versus 17.4%; *p* = 0.004; aOR = 6, 95% CI: 1.7–20.8, resp.) after adjusting for age and race (Table 2). 86% of participants with history of abdominal procedures stated that laparoscopy is better than open abdominal surgery compared with 52.7% of those who did not (*p* = 0.01; aOR = 5; 95% CI: 1.3–18.5).

Forty-six percent of participants did not understand the difference between laparoscopic and robotic-assisted laparoscopic procedures. A total of 67.5% of participants did not know that the surgeon moves the robot's arms to perform the surgery (Table 3). Moreover, despite the evidence that robotic-assisted laparoscopic procedure is more expensive than laparoscopic approach, only half of the participants (52.6%) believed that robotic-assisted laparoscopic surgeries cost more to the health care system than laparoscopic and open approaches [5, 7, 8]. Higher educational level and previous abdominal surgeries were associated with a significantly better understanding of how the surgeon performs robotic-assisted laparoscopic surgeries (*p* = 0.006;* p* = 0.006, resp.) (Table 3). Neither age nor race was related to how well participants understood the difference between conventional and robotic-assisted laparoscopic surgery.

When participants were asked to choose among the three available approaches if they were to undergo an abdominal surgery, 12% chose open approach and 33% had no preference. Before undergoing a surgery, 70% of participants would consult their physicians to discuss the best options. However, 22.4% of them would use the internet as their first source of information.

## 4. Discussion 

We have found that a majority of patients who seek OBGYN care are unaware of the differences between surgical approaches available to them. One might assume that advances in technology and information dissemination would have made it easier for patients to acquire such knowledge. Unfortunately, the quality of information and the ease with which it is obtained varies widely. Thus it is difficult for clinicians to predict what patients know about various surgical approaches, which increases the complexity of counseling and obtaining informed consent. It is important for surgeons to counsel their patients specifically about the risks and benefits of different surgical approaches.

In this study, only 66% of the surveyed patients understood the differences between open and laparoscopic surgery, with even fewer (54%) understanding the differences between laparoscopic and robotic-assisted laparoscopic surgery. Furthermore, more than half of the participants (54%) did not know that laparoscopic approach is associated with a less pain, shorter hospital stay, and faster recovery than open abdominal approach [2, 4]. Bucher et al. conducted two surveys to evaluate the population perception of minimally invasive procedures [9, 10]. Although the responders' main concern was the risk of surgical complications, they still favor scarless surgery (laparoendoscopic single-site surgery and natural orifice translumenal endoscopic surgery) over conventional laparoscopy. Similarly, Yeung et al. conducted a survey of 73 patients in two gynecologic clinics showing that most patients favor the incision of laparoendoscopic single-site surgery over the other types of abdominal surgery incisions [11]. In contrast, 45% of our participants did not elect to have their surgery done with laparoscopy or robotic-assisted laparoscopy. This elevated percentage correlates with their lack of knowledge of the difference between open abdominal surgery and laparoscopy (44%), which was significantly correlated with their level of education and history of previous abdominal surgery.

A survey of 241 women showed that they prefer both traditional and single-site laparoscopic incisions over robotic-assisted laparoscopic surgeries [12]. Likewise, a survey of 747 adults revealed that most respondents acknowledged the benefits of robotic-assisted laparoscopic surgeries but still preferred conventional laparoscopy [6]. Most of our participants (67.5%) did not know that the surgeon moves the robot's arms to perform the surgery confirming that their knowledge level of robotic-assisted laparoscopic procedures was even lower than that of conventional laparoscopic approaches. Such knowledge was also significantly correlated with their level of education and history of previous abdominal surgery. It is probable that participants who had faced the reality of undergoing surgery had researched their surgical options more thoroughly or had adequate counseling from their surgeons.

Some limitations of the current study should be mentioned. We did not investigate the patient's perception of vaginal approach in gynecologic procedures, which is considered the preferred minimally invasive approach in many situations [13]. Also, despite the identification of education and surgical history as factors that correlate with patient knowledge of various surgical approaches, it failed to identify the other contributing factors.

This study demonstrates that not all patients are aware of the differences in surgical approaches. Differences in education and surgical history may account for some of these differences, but not all. Health care providers may need to expand the time spent counseling their patients appropriately prior to any surgical procedure. This can be done via group information sessions, pamphlets, and brochures that can be made readily available in the office or via information that is displayed on the physician's website or blog [14, 15].

## Figures and Tables

**Table 1:** Demographic characteristics of survey respondents evaluating patient knowledge about surgical approaches in gynecology.

Characteristics	Number	Percentage
*Age (years)*		
18–29	101	46
30–59	110	50
>60	8	4
*Race*		
Caucasian	72	33
African American	40	18
Hispanic	43	20
Asian	47	22
Others	16	7
*Educational level*		
Less than high school	23	11
Some high school	77	35
Some college	68	31
College Graduate/professional	50	23
*Previous abdominal surgery*		
Yes	22	10
No	192	90

**Table 2:** Patient knowledge of laparoscopic and open abdominal surgeries. ^*∗*^ *p* < 0.05.

	Do not understand the difference between open and laparoscopic abdominal surgery *n* (%)	Laparoscopy is a better approach than open abdominal surgery *n* (%)	Higher skills are required for laparoscopic than open abdominal surgery *n* (%)
Race			
Caucasian	26 (36.1)	50 (69.4)	34 (47.2)
African American	11 (27.5)	23 (57.5)	14 (35)
Hispanic	20 (46.5)	16 (37.2)	11 (25.5)
Asian	16 (34)	19 (40.4)	14 (29.7)
Other	2 (12.5)	14 (87.5)	7 (43.7)
Age (years)			
<30	42 (41.5)	48 (47.5)	35 (34.6)
30–60	30 (27.2)	70 (63.6)	43 (39)
>60	3 (37.5)	5 (62.5)	3 (37.5)
Education			
<high school	13 (56.5)^*∗*^	3 (13)^*∗*^	4 (17.4)^*∗*^
High school degree	28 (36.3)	39 (50.6)	29 (37.6)
College degree	24 (35.2)	40 (58.8)	17 (25)
Professional	10 (20)	39 (78)	30 (60)
History of previous abdominal surgery			
Yes	0 (0)^*∗*^	19 (86.3)^*∗*^	12 (54.5)
No	75 (38)	194 (52.7)	69 (35)

**Table 3:** Patient knowledge of robotic and laparoscopic abdominal surgeries. ^*∗*^ *p* < 0.05.

	Do not understand the difference between robotic and laparoscopic abdominal surgery *n* (%)	Understand how robot works *n* (%)	Robotic surgeries are more expensive than laparoscopic or open abdominal surgery *n* (%)
Race			
Caucasian	28 (38.8)	29 (40.2)	37 (51.3)
African American	16 (40)	12 (30)	14 (35)
Hispanic	27 (63)	11 (25.5)	25 (58.1)
Asian	23 (49)	12 (25.5)	27 (57.4)
Other	7 (44)	7 (43.7)	11 (68.7)
Age (years)			
<30	52 (51.4)	25 (27.7)	53 (52.4)
30–60	48 (43.6)	42 (38.1)	57 (51.8)
>60	1 (12.5)	4 (50)	5 (62.5)
Education			
<high school	14 (60.8)	4 (17.3)^*∗*^	12 (52.1)
High school degree	33 (42.8)	20 (25.9)	43 (51.9)
College degree	35 (51.4)	21 (30.8)	36 (52.9)
Professional	19 (38)	26 (52)	23 (46)
History of previous abdominal surgery			
Yes	4 (18.1)^*∗*^	14 (63.6)^*∗*^	9 (40.9)
No	97 (49.2)	57 (28.9)	106 (53.8)

## Appendix

### Patient Survey Form

We are conducting a brief survey to assess attitudes and knowledge about various options for gynecologic surgery. Participation is entirely voluntary. All responses will remain anonymous.

What is your Age?
  — Years
What is your Education level?—Please check one of the following:
  □ less than high school
  □ some high school/high school degree
  □ some college/college degree
  □ graduate/professional degree
What is your race? (Please check one):
  □ Caucasian
  □ African American
  □ Hispanic
  □ Asian (Indian, Pakistani, Chinese, etc.)
  □ other — (please specify)
Have you had previous major surgery? (Do not count C-section or tonsil surgery)
  □ Yes
  □ No
  If Yes, what surgery(s)? —
(1) How well do you understand the differences between open abdominal surgery and laparoscopic surgery?
  I understand the difference:
  Not At All
  A Little
  Moderately
  Mostly
  Very Well
(2) In your opinion, which approach is better for patients? (Like less complications, faster recovery, shorter hospital stay, less pain, etc.). Check only one answer.
  □ Open abdominal surgery
  □ laparoscopic surgery
  □ both are the same
(3) In your opinion, which approach requires the surgeon to have a higher level of technical skill? Circle one answer choice.
  □ Open abdominal surgery
  □ Laparoscopic surgery
  □ both are the same
(4) How well do you understand the differences between laparoscopic and robotic surgery?
  I understand the
  difference:
  Not at all
  Little
  Somewhat
  pretty well
  Very well
(5) In Robotic surgery: (please choose the best answer)
  □ the surgeon moves the robot's arms to perform the surgery
  □ the surgeon tells the robot which surgery to perform, and the robot performs it while the surgeon supervises
  □ I do not know
(6) In your opinion, which surgery costs more to the health care system/insurance? Check only one answer.
  □ Open abdominal surgery
  □ laparoscopic surgery
  □ Robotic surgery
(7) If you were having surgery, which approach would YOU prefer? Circle only one answer choice.
  □ Open abdominal surgery
  □ Laparoscopic surgery
  □ Robotic surgery
  □ does not really matter
(8) If you needed surgery and you wanted to find out about the various options available, where would you first go for advice? (Pick one):
  □ Family member/friend
  □ Doctor
  □ Internet
